# Age‐Related Differences in Clinical Presentation of Autoimmune Gastritis: A Retrospective Comparative Study

**DOI:** 10.1155/grp/4250339

**Published:** 2025-12-11

**Authors:** Ren Song, Qi Chen, Xiao-Ling Wu, Ming Luo, Gang Hu

**Affiliations:** ^1^ Department of Gastroenterology, People’s Hospital of Dianjiang County, Dianjiang, Chongqing, China

**Keywords:** autoimmune gastritis, clinical manifestations, elder, gastroscopy finding, laboratory examinations

## Abstract

**Purpose:**

This study was aimed at investigating the influence of age on patients with autoimmune gastritis (AIG).

**Methods:**

A total of 56 patients diagnosed with AIG at our hospital between January 2019 and December 2021 were included in this study. The participants were categorized into the elderly group (≥ 60 years old) and the nonelderly group. We analyzed the baseline characteristics, gastroscopy findings, and various laboratory examination parameters to see if age significantly influences the clinical manifestation of AIG patients.

**Results:**

During the study period, 32 and 24 patients were included in the elderly and nonelderly groups. Regarding baseline characteristics and symptoms, the nonelderly group showed a higher prevalence of acid reflux (12.5% vs. 6.3%, *p* < 0.05), a higher proportion of asymptomatic patients (8.3% vs. 3.1%, *p* = 0.046), and a higher prevalence of iron deficiency anemia (37.5% vs. 12.5%). Regarding laboratory examinations, the nonelderly group had a lower mean corpuscular volume (78.8 ± 7.9 vs. 89.2 ± 8.1 fL, *p* = 0.024), decreased serum ferritin levels ((24.8 ± 10.9 vs. 48.4 ± 13.1 ng/mL, *p* = 0.024), elevated serum vitamin B12 (92.3 ± 18.2 vs. 76.8 ± 12.9 pmol/L, *p* = 0.037), and a higher incidence of positive thyroid peroxidase antibody (28.2% vs. 12.5%, *p* = 0.024). However, the gastroscopy findings, including the incidence of proliferative polyps, neuroendocrine tumors, gastric intraepithelial neoplasia, and cancers, showed no significant difference between the two groups (*p* > 0.05).

**Conclusion:**

Nonelderly patients with AIG exhibit distinct clinical features compared to elderly patients. Large sample sizes with multiple centers involved in studies are required to verify our findings.

## 1. Introduction

Autoimmune gastritis (AIG) is a chronic inflammation and atrophy of the gastric fundus caused by autoimmunity. It can lead to various clinical symptoms such as indigestion, anemia, nerve damage, and complications such as endocrine tumors and gastric cancer (GC) [1–3]. These changes are due to the antiparietal‐cell antibodies, which may destroy gastric parietal cells in AIG [4].

Patients with AIG have a three‐ to fivefold increased risk of developing GC [5]. A recent study suggests that AIG may replace *Helicobacter pylori* as the primary driver of GC risk [4, 6]. Therefore, regular monitoring of AIG patients through gastroscopy is recommended [7].

In the past, AIG was commonly observed in elderly individuals. However, in recent years, there has been an increase in nonelderly AIG patients due to improved diagnostic awareness, diagnostic capabilities, and high‐resolution endoscopy [8]. The clinical features of AIG in nonelderly patients may differ from those in elderly patients due to differences in gastrointestinal physiology. Studying and comparing the clinical characteristics of AIG across different age groups can enhance diagnostic accuracy and prevent delays and misdiagnosis; however, relevant studies are lacking.

Therefore, this study is aimed at compare the clinical manifestations, laboratory examinations, and gastroscopy results between elderly and nonelderly individuals with AIG, thereby exploring the similarities and differences between these two groups.

## 2. Materials and Methods

### 2.1. Patient Information and Inclusion Criteria

The retrospective collection of patients with AIG diagnosed at Dianjiang County′s People Hospital in Chongqing, China, from January 2019 to December 2021 involved the following criteria. Both of the following conditions validated the AIG diagnosis: severe mucosal atrophy, predominantly from the gastric body to the fundus, based on endoscopic and/or histological findings, and positivity for gastric autoantibodies using PCA and/or IFA tests. The criteria were consistent with the principles proposed by Livzan et al. [9]. Exclusion criteria: (1) cases with total gastric retention reported during gastroscope or incomplete observation, (2) individuals previously diagnosed with AIG and undergoing follow‐up as per medical advice, and (3) individuals with no complete medical records.

## 3. Methods

All patients who participated in this study had abstained from taking acid suppressants for at least 1week before the examination. The laboratory examination encompassed routine blood examination; the following indicators were obtained and analyzed: white blood cell count (WBC), red blood cell count (RBC), platelet count (PLT), hemoglobin (Hb) level, mean corpuscular volume (MCV), and mean corpuscular hemoglobin concentration (MCHC) levels. Other laboratory examinations were also measured and analyzed, including folate, vitamin B12, ferritin, gastrin‐17, pepsinogen I, parietal cell antibodies, and endogenous factor antibodies. The antibody titer was automatically determined using a chemiluminescent enzyme immunoassay (CLEIA), with a value of 20.0 U/mL defined as positive, according to the manufacturer′s instructions. Gastroscopy biopsies were obtained from at least two sites (one from the gastric antrum, one from the gastric body, and potentially one additional sample from the gastric horn or a pathological tissue). The extent of gastric mucosa atrophy, the presence or absence of intestinal metaplasia, and pyloric gland metaplasia were assessed. The representative images are shown in Figure 1. The slight cell hypopigmentation anemia was defined as decreased Hb: male < 120 g/L, female < 110 g/L, MCV < 80 fL, mean corpuscular hemoglobin (MCH) < 27 pg, and MCHC < 320 g/L. Megaloblastic anemia was defined as decreased Hb: male < 120 g/L, female < 110 g/L, MCV > 100 fL, and MCH > 32 pg. Other possible confounders such as dietary iron/B12 intake and menstrual status in younger women were excluded.

Figure 1Representative figure of autoimmune gastritis. (a, b) A 52‐year‐old female patient. (a) Gastroscopy revealed extensive atrophy of the gastric mucosa, accompanied by the presence of micropolyps. (b) Pathology showed atrophic gastritis and lymphoid follicular hyperplasia. (c, d) A 68‐year‐old female patient. (c) Gastroscopy revealed extensive atrophy of the gastric mucosa, accompanied by hyperplasia of the mucosa at the greater curvature in the middle and lower parts. (d) Pathology showed high‐grade intraepithelial neoplasia.

**Figure:**
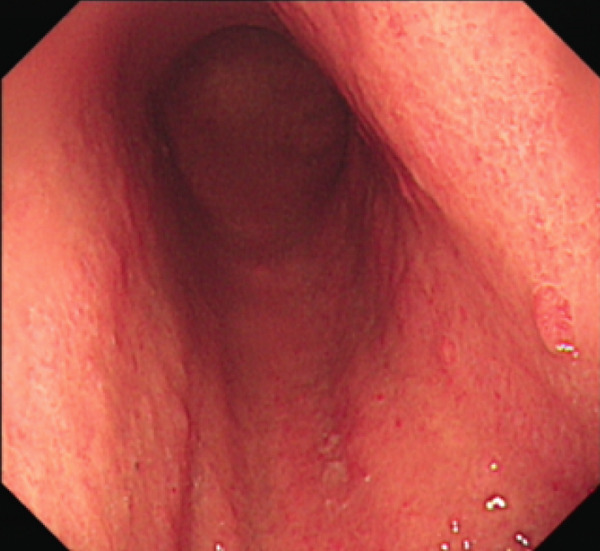
(a)

**Figure:**
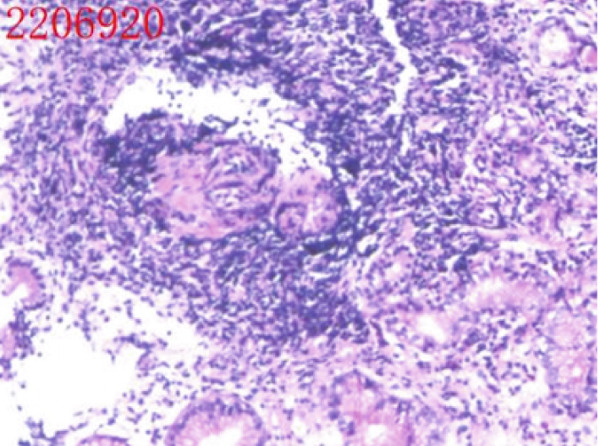
(b)

**Figure:**
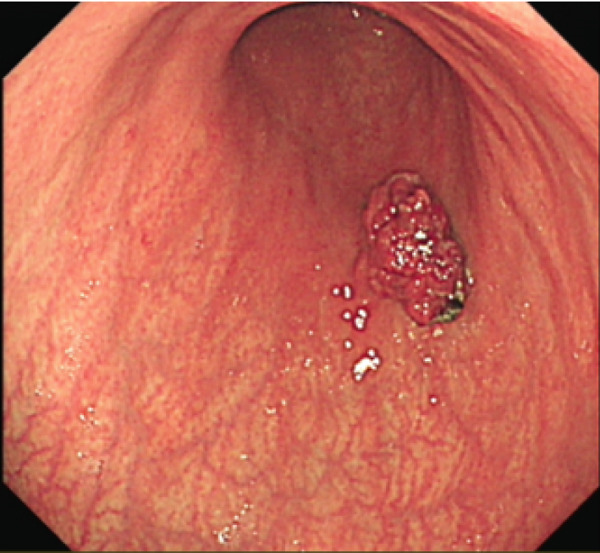
(c)

**Figure:**
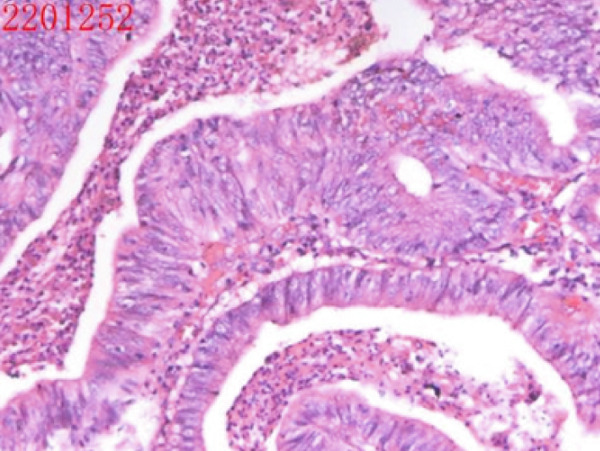
(d)

### 3.1. Statistical Methods

Data analysis was performed using SPSS19.0 statistical software. Measurement data were presented as mean ± standard deviation (mean ± SD). The two groups were compared using *t*‐tests; counting data were expressed as rates, and intergroup comparisons were performed using *χ* ^2^ tests. The Benjamini–Hochberg correction was performed to adjust the multiple‐testing *p* value and calculate the false discovery rate (FDR). A significance level of *p* < 0.05 was used to determine statistical significance.

## 4. Results

### 4.1. General Information

In the current study, a total of 56 AIG patients were included, aged from 33 to 82 years, with an average age of 60.8 ± 18.9 years. Among them, nine were male (16.1%). There were 32 elderly patients (57.1%), with a cutoff age of 60 years, and the average age was 71.8 ± 8.6 years. Among the elderly patients, 26 were female (81.3%). There were 24 patients (42.9%) in the nonelderly group, with an average age of 49.8 ± 10.3 years, and 21 of them were female (87.5%). There was no statistically significant difference in gender distribution between the two patient groups (*p* = 0.127).

### 4.2. Clinical Features of Elderly and Nonelderly AIG Patients

Our department conducted 30,646 gastroscopy examinations during the investigation period, with 6418 cases undergoing re‐examination. The actual number of gastroscopy visits was 24,196 times. Among them, 56 cases were diagnosed as AIG based on pathological outcomes and laboratory examinations, resulting in an incidence of AIG of 0.2%.

In the elderly group, clinical symptoms persisted for 2–10 years. Among these cases, except for one patient diagnosed as AIG by asymptomatic gastroscopy, the remaining 26 cases (81.3%) experienced abdominal distension. Three cases (9.4%) reported fatigue, and two cases (6.3%) had acid reflux (Table 1). Three cases showed anemia, three were found to have *H. pylori* eradication failures, and three had concomitant malignant tumors.

**Table 1:** Demography and clinical characteristics of elderly and non‐elderly AIG patients.

**Clinical characteristics**	**Elderly (** **n** = 32**)**	**Nonelderly (** **n** = 24**)**	**p** **value**
Age (years)	71.8 ± 8.6	49.8 ± 10.3	
Gender (male/female)	6/26	3/21	0.127
Clinical symptoms			
Abdominal distension	26 (81.3%)	17 (70.8%)	0.352
Fatigue	3 (9.4%)	2 (8.3%)	0.114
Acid reflux	2 (6.3%)	3 (12.5%)	0.114
Asymptomatic	1 (3.1%)	2 (8.3%)	**0.046**
Diagnostic clues			
Gastroscope	27 (84.4%)	17 (70.8%)	0.798
Anemia	29 (90.6%)	13 (54.2%)	**0.004**
*H. pylori* eradication failure	2 (6.3%)	3 (12.5%)	0.114
Thyroid disease	0 (0.0%)	2 (8.3%)	‐ ‐
Cases of concomitant malignant tumor	3 (6.3%)	0 (0.0%)	

*Note:* Bold indicates *p* < 0.05.

In the nonelderly group, except for two cases (8.3%) diagnosed with asymptomatic AIG during the autoimmune thyroid monitoring period, 17 cases (70.8%) presented with abdominal distension, two cases (8.3%) experienced fatigue, and two cases (12.5%) reported acid reflux, with a duration of symptoms ranging from 6 months to 5 years. Two cases (8.3%) showed anemia, three cases (12.5%) had *H. pylori* eradication failures, and two cases (8.3%) had concomitant thyroid diseases (Table 1).

### 4.3. Gastroscopy and Laboratory Examination

All participants underwent gastroscopy examinations, and the results showed that all patients exhibited solid and turbid mucus under the macroscopic examination. In all cases, we also observed diffuse atrophy of the gastric fundus gland area (including the gastric body and fundus) with a normal gastric antrum.

In the elderly group, gastroscopy findings provided diagnostic clues in 27 cases (84.4%) (gastric mucosal atrophy). Then, 13 cases (40.6%) were confirmed to have red nodular proliferative polyps through biopsy, and four cases (12.5%) had neuroendocrine tumors. In addition, two cases (6.3%) showed early GC, including one with high‐grade intraepithelial neoplasia and one with local intramucosal carcinogenesis. Meanwhile, laboratory examinations revealed that 25 participants (78.1%) had megaloblastic anemia, and four patients (12.5%) had slight cell hypopigmentation anemia. Moreover, vitamin B12 levels were decreased in 30 patients (93.8%), serum iron levels were decreased in 17 cases (53.1%), parietal cell antibody was positive in 30 cases (93.8%), endogenous factor antibody was positive in six cases (18.8%), and thyroid peroxidase antibody was positive in four cases (12.5%). Furthermore, regarding the C14 breath test, 11 cases (34.4%) showed negative results, and 21 cases (65.6%) had a critical value (Table 2).

**Table 2:** Outcomes of the gastroscopy and laboratory examination for elderly and nonelderly AIG patients.

**Project**	**Elderly group (** **n** = 32**)**	**Nonelderly group (** **n** = 24**)**	***p* value**	**FDR** ^**a**^
Gastroscope				
Proliferative polyps	13 (40.6%)	5 (20.8%)	**0.013**	**0.0900**
G‐NETs	4 (12.5%)	3 (12.5%)	0.167	0.2610
Intraepithelial neoplasia	2 (6.3%)	2 (8.3%)	**0.041**	**0.1025**
Blood parameters				
RGB (×10^12^/L)	3.2 ± 1.3	3.4 ± 1.1	0.313	0.3718
Hb (g/L)	89.7 ± 22.7	98.6 ± 29.7	0.451	0.4510
MCV (fL)	89.2 ± 8.1	78.8 ± 7.9	**0.024**	**0.0900**
WBC (×10^9^/L)	5.8 ± 3.5	5.2 ± 2.9	0.347	0.3718
PLT (×10^9^/L)	177.8 ± 48.9	186.2 ± 57.1	0.347	0.3718
Ferritin (ng/mL)	48.4 ± 13.1	24.8 ± 10.9	**0.014**	**0.0900**
VitaminB12 (pmol/L)	76.8 ± 12.9	92.3 ± 18.2	**0.037**	**0.1025**
PG‐I (*μ*g/L)	14.8 ± 5.3	15.5 ± 6.8	0.326	0.3718
IG‐17 (pmol/L)	64.3 ± 12.1	61.8 ± 15.6	0.129	0.2419
PCA (+)	30 (93.8%)	23 (95.8%)	0.068	0.1457
IFA (+)	6 (18.8%)	5 (20.8%)	0.174	0.2610
Thyroid peroxidase antibody	4 (12.5%)	7 (28.2%)	**0.024**	**0.0900**

*Note:* Bold indicates *p* < 0.05.

Abbreviations: FDR, false discovery rate; Hb, hemoglobin; IFA, intrinsic factor antibody; IG‐17, gastrin‐17; MCV, mean corpuscular volume; PCA, parietal cell antibody; PG‐I, pepsinogen I; PLTs, platelets; RGB, red blood cells; WBC, white blood cells.

^a^Benjamini–Hochberg correction was used.

In the nonelderly group, endoscopic findings suggested AIG in 17 cases (70.8%) (gastric mucosal atrophy), proliferative polyps in five cases (20.8%), neuroendocrine tumors in three cases (12.5%), and early GC in two cases (8.3%). The laboratory examinations indicated that four cases (16.7%) had megaloblastic anemia and nine cases (37.5%) had slight cell hypopigmentation anemia. Additionally, six patients (25.0%) exhibited decreased vitamin B12 levels, 11 patients (45.8%) had decreased serum iron levels, 23 patients (95.8%) tested positive for parietal cell antibody, five individuals (20.8%) were positive for endogenous factor antibody, and seven cases (28.2%) were positive for thyroid peroxidase antibody. Similar to the elderly group, 11 participants (45.8%) had negative results in the C14 breath test, while 13 cases (54.2%) had a critical value (Table 2).

### 4.4. Comparison Results of Parameters Between Elder and Nonelder Patients

Regarding baseline characteristics and symptoms, the nonelderly group showed a higher prevalence of acid reflux (12.5% vs. 6.3%, *p* < 0.05), a higher percentage of asymptomatic patients (8.3% vs. 3.1%, *p* = 0.046), and a higher prevalence of iron deficiency anemia (37.5% vs. 12.5%). Regarding laboratory examinations, the non‐elderly group had a lower MCV (78.8 ± 7.9 vs. 89.2 ± 8.1 fL, *p* = 0.024), decreased serum ferritin levels (24.8 ± 10.9 vs. 48.4 ± 13.1 ng/mL, *p* = 0.024), elevated serum vitamin B12 (92.3 ± 18.2 vs. 76.8 ± 12.9 pmol/L, *p* = 0.037), and a higher incidence of positive thyroid peroxidase antibody (28.2% vs. 12.5%, *p* = 0.024). However, regarding the gastroscopy findings, there was no significant difference between the two patient groups (*p* > 0.05), including the incidence of proliferative polyps, neuroendocrine tumors, gastric intraepithelial neoplasia, and cancers.

## 5. Discussion

AIG was previously considered to be an organ‐specific autoimmune inflammatory disease, which tended to occur in elderly patients, but is now increasingly detected in nonelderly populations [1, 2]. In this study, for the first time, we compared the clinical symptoms, gastroscopy findings, and laboratory examination outcomes between the elderly and nonelderly AIG patients, revealing that iron deficiency anemia and autoimmune thyroid diseases are more prevalent among nonelderly patients. The findings suggest that nonelderly individuals with AIG, especially those with autoimmune thyroid diseases, should undergo early screening for gastric parietal cell antibodies, intrinsic factor antibodies (IFAs), and other relevant biomarkers to facilitate early detection and promote an earlier diagnosis of AIG. This approach represents a novel shift from tertiary prevention (diagnosing advanced atrophy) to secondary prevention (early detection, diagnosis, and treatment), emphasizing the importance of targeted screening in the non‐elderly population. By implementing this strategy, clinicians can enhance the early identification of AIG, thereby improving patient outcomes through timely intervention.

One notable aspect of AIG is the lack of specificity in the clinical symptoms [10]. In a cross‐sectional study involving 379 AIG patients, 69.8% reported abdominal fullness and indigestion as the primary manifestations, with post‐meal satiety being a common symptom, particularly in nonelderly individuals. Additionally, 36.5% of patients experienced fatigue [1]. In our study, abdominal distension and fatigue were observed in 81.3% and 9.4% of the elderly group and 70.8% and 8.3% of the non‐elderly group, respectively. The most prevalent symptom was postprandial upper abdominal distension. The results were consistent with previous literature [1]. Notably, the nonelderly group exhibited a significantly higher incidence of acid reflux (12.5%) than the elderly group (6.3%), with some cases showing prominent symptoms. Meanwhile, Carabotti et al. [11] reported that 54 AIG patients exhibited typical symptoms of gastroesophageal reflux in 24%, upper abdominal heartburn in 9.2%, and acid reflux in 18.5%. Therefore, we can conclude that although a lack of sufficient digestive ability is the predominant symptom of AIG, some patients still experience the symptoms of excessive acid.

Gastric acid was inhibited in AIG patients due to the destruction of parietal cells. Typically, gastric acid and pepsin facilitate the chemical digestion of proteins and fats [12]. However, in AIG, reduced gastric acid secretion and pepsin levels are associated with impaired gastric chemical digestion, weakened gastroduodenal peristalsis, delayed gastric emptying, and increased gastroesophageal reflux and duodenogastric reflux [12–15]. This may be the reason why AIG patients have excess acid symptoms. Tenca et al. [16] found that among 41 AIG patients monitored with esophageal pH impedance, only 10 showed low acid and/or base reflux, indicative of hypochlorhydria. Similarly, Kalkan et al. [13] investigated 165 AIG patients using gastric emptying scintigraphy. They found that 80% of the patients had delayed gastric emptying, further supporting our findings. Therefore, when encountering nonelderly patients with symptoms suggestive of long‐standing acid reflux accompanied by anemia symptoms, it is crucial to consider the possibility of AIG‐induced low gastric acid, impaired pepsin digestion, and delayed gastric emptying. This approach helps avoid the indiscriminate use of proton pump inhibitors for gastroesophageal reflux disease, which may delay the diagnosis of AIG.

Additionally, in laboratory examinations related to AIG, the presence of parietal cell antibodies and IFAs is a significant indicator, including those associated with iron deficiency anemia and megaloblastic anemia. Recent evidence revealed that megaloblastic anemia accounted for 53.6%, and iron deficiency anemia accounted for 34.8% of the investigated 373 AIG cases [11]. Iron deficiency anemia is more common in young AIG patients, whereas megaloblastic anemia is more prevalent in the elderly [17]. Long‐term follow‐up revealed that some cases progress from iron deficiency anemia to megaloblastic anemia [15]. In our study, 29 cases (90.6%) in the elderly group and 13 cases (59.1%) in the nonelderly group presented with anemia, showing a statistically significant difference. Moreover, when classifying the anemia types into megaloblastic anemia and small cell hypopigmentation anemia, the elderly group had 25 cases (78.1%) and 4 cases (12.5%), respectively, whereas the nonelderly group had four cases (16.7%) and nine cases (37.5%), respectively. Megaloblastic anemia is more prevalent in the elderly group, while small cell hypopigmentation anemia is more common in the nonelderly group. Furthermore, laboratory examination results demonstrated that the MCV in the elderly group was 89.2 ± 8.1 fL, whereas in the nonelderly group, it was 78.8 ± 7.9 fL, demonstrating a significant difference. In addition, the nonelderly group exhibited lower serum ferritin levels associated with anemia. All the results indicated that nonelderly patients suffered from more iron deficiency than elderly patients, which differs from a previous study [18].

AIG often coexists with other autoimmune diseases, similar to common autoimmune conditions. Reports indicated that 24%–35% of AIG patients may be contaminated with autoimmune thyroid diseases, particularly Hashimoto′s thyroiditis. The presence of thyroid peroxidase and other related indicators may be detected in these patients [14, 19]. In addition to autoimmune thyroid disease, other conditions, such as rheumatoid arthritis, vitiligo, and psoriasis, are relatively rare. In this study, four cases (12.5%) in the elderly group and seven (28.2%) in the nonelderly group tested positive for thyroid peroxidase antibodies. This suggests a higher prevalence of positive thyroid peroxidase antibodies in the nonelderly groups compared to the elderly group. Importantly, two cases (8.3%) had autoimmune thyroid disease as the initial clue, serving as the sole indication for AIG. The association between AIG and immune thyroiditis is further supported by the shared embryonic origin of the thyroid and stomach, known as “thyroid gastric syndrome” [1, 12, 20, 21].

Finally, gastroscopy plays a crucial role in diagnosing AIG, as various gastroscopic signs such as gastric mucosal atrophy, proliferative polyps, gastric neuroendocrine tumors (G‐NETs), intraepithelial neoplasia, and pathological mucus have become diagnostic indicators for AIG [2]. In this study, the prevalence of proliferative polyps in the elderly group (40.6%) was significantly higher than in the nonelderly group (20.8%), which is consistent with the findings of the AGAPE study [22]. These polyps manifest as red nodular hyperplasia during gastroscopy and can be confirmed through pathological examination.

The progression of AIG involves three pathomorphological stages: the early phase, the florid phase, and the end‐stage. During the early stage, gastroscopy may not reveal any specific changes. However, as the disease progresses, parietal cells are continuously attacked by the immune system, leading to extensive atrophy of gastric fundus glands, intestinal metaplasia, pyloric metaplasia, and other changes. The gastric antrum mucosa is generally normal or slightly abnormal, while the gastric fundus and gastric body exhibit atrophy, referred to as “reverse atrophy” [14, 23]. Meanwhile, immune‐mediated damage to parietal cells results in reduced gastric acid and endogenous factors production [24]. Also, the decreased gastric acid feedback stimulates the proliferation of G cells in the gastric antrum. This excessive release of gastrin promotes the proliferation or dysplasia of enterochromaffin cells in the stomach and intestines, which may progress to type I G‐NETs [2, 14]. In our study, there was no significant difference between the two groups compared to AIG in terms of AIG‐related intraepithelial neoplasia and type I G‐NETs. No significant differences were observed between the two groups, consistent with a previous study [20].

Gastroscopy screening is a critical diagnostic approach for AIG, with common microscopic signs including solid and turbid mucus, scattered small white protrusions, and retrograde atrophy. In our study, 42.3% of AIG patients exhibit varying degrees of gastric antral atrophy, indicating total gastric atrophy involving the gastric fundus and pyloric glands. In addition, magnified gastroscopy observation can aid in the diagnosis of AIG by detecting mucosal atrophy and enlarged small white protrusions. The present study is associated with several limitations. The sample size of this study was limited, which might require more sample sizes for verification. The study was conducted at a single medical center, and most participants were socially active and productive. A comparative study is thus needed to clarify the difference in the prevalence of AIG among the different regional dietary and genetic populations.

## 6. Conclusions

In summary, elderly and nonelderly AIG patients exhibit distinct clinical characteristics. Nonelderly AIG patients often experience abdominal discomfort and iron deficiency anemia. Our study may offer a novel approach to the accurate diagnosis and timely treatment of AIG.

**Nomenclature**
AIG: autoimmune gastritis
WBC: white blood cell count
RBC: red blood cell count
PLT: platelet count
Hb: hemoglobin
MCV: mean corpuscular volume
MCHC: mean corpuscular hemoglobin concentration
PCAs: parietal cell antibodie
IFAs: intrinsic factor antibodies
G‐NETs: gastric neuroendocrine tumors

## Ethics Statement

This study was conducted according to the guidelines of the Declaration of Helsinki and approved by the Ethics Committee of People′s Hospital Dianjiang County. Because this is a retrospective clinical data analysis, the subjects′ written informed consents were waived by the Ethics Committee of People′s Hospital Dianjiang County.

## Consent

The authors have nothing to report.

## Disclosure

All authors read and approved the final version of the manuscript.

## Conflicts of Interest

The authors declare no conflicts of interest.

## Author Contributions

Ren Song conceived and designed the research, wrote the initial draft and revised the manuscript, and was primarily responsible for the final content. Qi Chen and Xiao‐Ling Wu collected data and conducted research. Ming Luo and Gang Hu analyzed and interpreted data.

## Funding

No funding was received for this manuscript.

## Data Availability

The datasets generated and analyzed during the current study are available from the corresponding author upon reasonable request.
